# A stable isotope dilution method for a highly accurate analysis of karrikins

**DOI:** 10.1186/s13007-021-00738-1

**Published:** 2021-04-01

**Authors:** Jakub Hrdlička, Tomáš Gucký, Johannes van Staden, Ondřej Novák, Karel Doležal

**Affiliations:** 1 Laboratory of Growth Regulators, Institute of Experimental Botany, The Czech Academy of Sciences and Faculty of Science, Palacký University, Šlechtitelů 27, 78371 Olomouc, Czech Republic; 2 Department of Chemical Biology, Faculty of Science, Palacký University, Šlechtitelů 27, 78371 Olomouc, Czech Republic; 3 Department of Experimental Biology, Faculty of Science, Palacký University, Šlechtitelů 27, 78371 Olomouc, Czech Republic; 4 Research Centre for Plant Growth and Development, School of Life Sciences, University of KwaZulu-Natal Pietermaritzburg, Private Bag X01, Scottsville, 3209 South Africa

**Keywords:** Karrikins, Smoke water, Stable isotope labelled standard, Stable isotope dilution method, Ultra-high performance liquid chromatography (UHPLC), Tandem mass spectrometry (MS/MS)

## Abstract

**Background:**

Karrikins (KARs) are recently described group of plant growth regulators with stimulatory effects on seed germination, seedling growth and crop productivity. So far, an analytical method for the simultaneous targeted profiling of KARs in plant tissues has not been reported.

**Results:**

We present a sensitive method for the determination of two highly biologically active karrikins (KAR_1_ and KAR_2_) in minute amounts of plant material (< 20 mg fresh weight). The developed protocol combines the optimized extraction and efficient single-step sample purification with ultra-high performance liquid chromatography-tandem mass spectrometry. Newly synthesized deuterium labelled KAR_1_ was employed as an internal standard for the validation of KAR quantification using a stable isotope dilution method. The application of the matrix-matched calibration series in combination with the internal standard method yields a high level of accuracy and precision in triplicate, on average bias 3.3% and 2.9% RSD, respectively. The applicability of this analytical approach was confirmed by the successful analysis of karrikins in *Arabidopsis* seedlings grown on media supplemented with different concentrations of KAR_1_ and KAR_2_ (0.1, 1.0 and 10.0 µmol/l).

**Conclusions:**

Our results demonstrate the usage of methodology for routine analyses and for monitoring KARs in complex biological matrices. The proposed method will lead to better understanding of the roles of KARs in plant growth and development.

**Supplementary Information:**

The online version contains supplementary material available at 10.1186/s13007-021-00738-1.

## Background

**Fig. 1:**
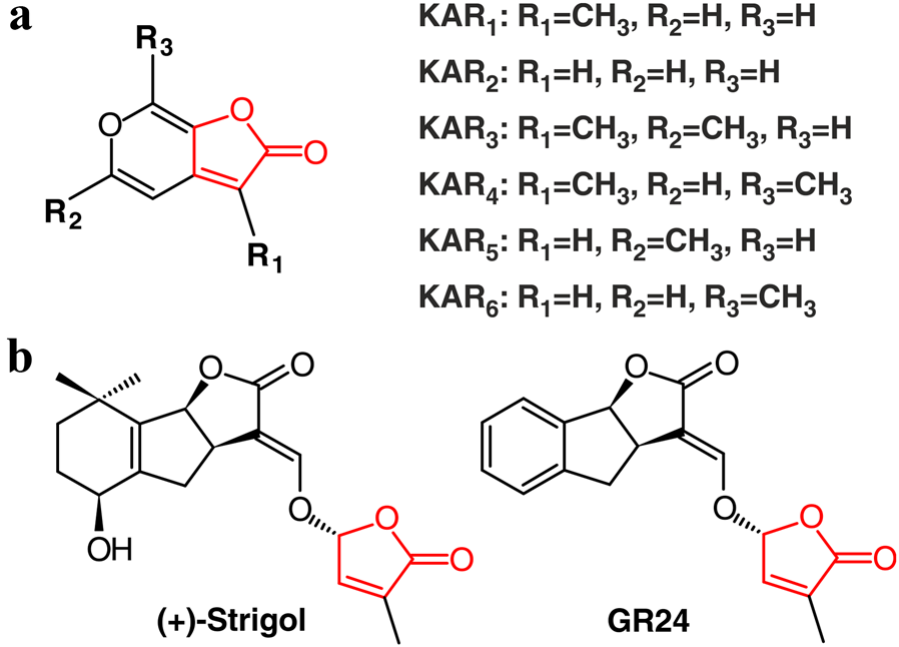
The structures of six naturally occurring karrikins (**a**) and strigolactones (**b**). The structural similarity of KARs and SLs, naturally occurring strigol and synthetic analogue GR24, is due to the presence of a 2-furanone moiety (red part)

Karrikins (KARs) are small organic compounds derived from butenolide molecules (Fig. 1a) with their principal effect on seed germination [1, 2]. Details of their origin is still unknown, but their formation requires oxygen and a pyran ring derived from the heating of polysaccharides and sugars [3, 4]. KARs are produced during wild fires and play a key role in the restoration of destroyed areas. Moreover, they can also be components of artificially prepared saturated extracts (so-called smoke water, SW). The SW prepared from smoke produced by controlled combustion devices can be used as an affordable biostimulant in agriculture and horticulture [2, 5, 6]. The stimulatory effect of KARs is independent from plant reproductive strategy, seed size and/or plant morphology or ecology. They are effective not only on plants from fire-prone areas, but also on various species from different families and environments [5]. There are six known KARs (Fig. 1a), of which KAR_1_ is most active on seed germination of fire-following plants, but the model plant *Arabidopsis* responds more strongly to KAR_2_ [7]. In parallel, a structurally and functionally similar substance, named strigolactone (SL, Fig. 1b), has been shown to trigger germination of parasitic plants [8, 9]. KARs are perceived by KARRIKIN-INSENSITIVE2 (KAI2), a homolog of SLs receptor. In addition, both signaling pathways share the same or similar components, such as the F-box protein MORE AXILARY GROWTH2 (MAX2) and the transcriptional corepressors SUPPRESSOR OF MAX2 1 (SMAX1)-LIKE proteins (SMXL) [10, 11].

A better understanding of plant growth regulators’ (PGRs) biosynthesis, metabolism and mode of action requires analysis of changes in their levels in various plant organs and tissues in different physiological processes. Moreover, the biological activity of PGR compounds is dependent on their concentration levels both in the whole plant and in individual organs. It is therefore of great importance to develop highly sensitive and robust analytical methods for monitoring the endogenous levels of PGRs in various plant tissues. Such analyses still remain challenging, since PGRs are present at very low concentrations (pmol/g fresh weight) in a complex biological matrix [12]. Typical analysis of plant hormones usually includes a multi-step sample pre-treatment, such as solid phase extraction (SPE) and/or immunoaffinity extraction followed by subsequent instrumental measurements of individual metabolites [13]. SPE is the most frequently used method to isolate compounds from complex matrices. By removing interfering compounds such as salts, pigments, polysaccharides, lipids and proteins from the sample, the chemical noise is reduced and potential co-eluting analytes are eliminated. In addition, less complex samples are more gentle on highly sensitive instrumentation such as mass spectrometers.

During the last decade, liquid chromatography (LC) or gas chromatography (GC) coupled to tandem mass spectrometry (MS/MS) has been widely used to determine levels of various plant hormones [reviewed in 12–17]. The complexity of plant matrix, where minor PGRs are present in the background of more abundant primary and secondary metabolites, requires a combination of proper sample preparation and high instrument performance (robustness and sensitivity). For assessment of the accuracy and precision of MS-based methods, the concentration of each analyte should be calculated using the stable isotope dilution method (SIDM; [16]). In general, SIDM is used to determine the quantity of a chemical substance in a sample based on internal standard (IS) to analyte ratio. Moreover, IS labelled with stable isotopes such as deuterium (^2^H), ^13^C, ^15^ N, and/or ^18^O atoms balances the inefficiencies and/or losses within the process of sample preparation as well as the ion suppression effects during the MS analysis [18]. In the case of using deuterium-labelled IS, three or more ^2^H are used. One reason is the stronger binding of ^2^H isotopes to carbons than ^1^H isotopes, which can lead to small physico-chemical differences between the analyte and IS and thus potentially better chromatographic separation. In addition, a higher number of ^2^H prevents interferences of naturally occurring analyte isotopes with IS [19]. In plant hormone profiling methods, the use of IS for targeted quantification analysis has become the primarily performed technique [showed in 20–23].

Very recently, ultra-high performance liquid chromatography combined with tandem mass spectrometry (UHPLC–MS/MS) was used for KAR quantification [24]. A standard dilution method (SDM) using a KAR structural analogue and a standard addition method (SAM) were compared. The SAM was successfully validated and applied in the determination of KARs in eight smoke water samples of various origins and ages. However, the SIDM was not tested due to the absence of an isotopically labelled standard. In this study, we have developed a complex analytical protocol suitable for the SPE-based isolation of KARs supplemented by sensitive and selective quantification using the UHPLC–MS/MS method. After synthesizing a new deuterium-labelled internal standard, we were able to apply the SIDM as a quantification approach for KAR analyses. Finally, we demonstrated utility of our MS-based method for KARs profiling in samples of ten-day-old seedlings of *Arabidopsis thaliana* treated with KAR_1_ and KAR_2_, which are commercially available and commonly used in plant research.

## Results and discussion

### Preparation of stable isotope labelled standard [^2^H_3_]KAR_1_

A previously published study showed the difficulties of KAR quantification due to the complexity of smoke water matrices [24]. Although the SAM method has been validated, its application for the analysis of biological samples is time consuming [25]. Moreover, the methodology can be less suitable for tissue specific experiments due to the requirement for a large amount of plant material. The targeted profiling of KARs in minute plant tissues using a new deuterium-labelled internal standard was an essential requirement in the field of karrikin research. Therefore, the development of a stable isotope dilution method was initiated by the synthesis of a new IS.

**Fig. 2:**
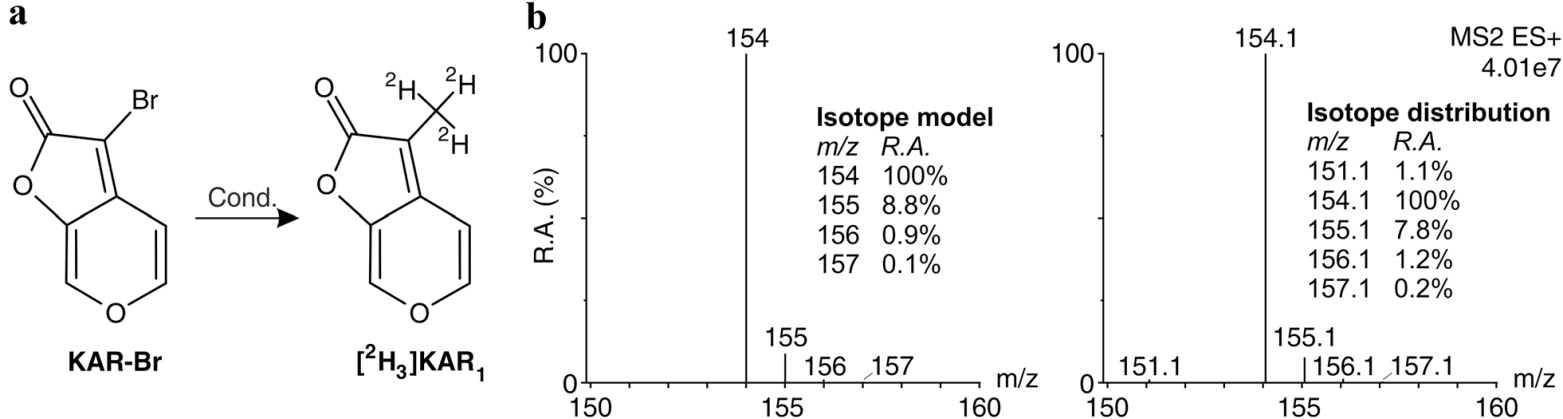
Preparation scheme and isotope distribution of a newly synthesized internal standard. **a** Stable isotope labelled standard ([^2^H_3_]KAR_1_) was prepared from KAR bromoderivative (KAR-Br) under the following conditions (Cond.): [^2^H_3_]CB(OH)_2_, Pd(OAc)_2_, S-Phos, K_3_PO_4_, toluene. **b** Full-scan (*m/z* 150–160) positive-ion mass spectrum of [^2^H_3_]KAR_1_ (right), theoretical isotope model of [^2^H_3_]KAR_1_ (Molecular Formula: C_8_H_3_D_3_O_3_, left). R.A. – relative abundance (%)

The isotopically labelled 3-(^2^H_3_)methyl-2H-furo[2,3-c]pyran-2-one ([^2^H_3_]KAR_1_) was prepared from 3-bromo-2H-furo[2,3-c]pyran-2-one (KAR-Br) by the coupling reaction with trideuteromethylboronic acid (Fig. 2a). The reaction was performed analogously to the previously reported preparation of 3-methyl-2H-furo[2,3-c]pyran-2-one (KAR_1_) [26]. We did not use a greater excess of trideuteromethylboronic acid due to its limited availability and probably for this reason the yield of the reaction was significantly decreased by the formation of side product, furo[2,3-c]pyran-2-one, in approximately 35% yield. The crude product was then purified twice by column chromatography. The identity and purity of prepared [^2^H_3_]KAR_1_ was examined using ^1^H and ^13^C NMR spectrometry, HPLC–DAD-(ESI +)MS and GC-(EI)MS methods. The isotopic purity was calculated from LC–MS data of the product after subtraction of a theoretical isotope model (Fig. 2b). The molar content of (^2^H_3_]KAR_1_) was at least 99.0%. However, the content of non-deuterated KAR_1_ was also detected, not more than 1.0%. Importantly, dideutero- and monodeutero-derivatives were not detected (Fig. 2b). The isotopic purity is sufficient for the proposed use of the newly synthesized compound ([^2^H_3_]KAR_1_) as an internal standard for the quantification of karrikins in various (non-)biological materials.

### Development of extraction and purification protocol

Due to the expected extremely low concentration of KARs in plants, it has been necessary to develop an efficient extraction step with a suitable solvent under optimized conditions. The intriguing similarity between two germination stimulants, karrikins and strigolactones (Fig. 1) inspired us to develop extraction approaches for KARs isolation from complex plant matrices. Both groups of substances are soluble in water and/or in mixtures of semi-polar organic solvents [24, 27]. This feature can be used to advantage in the extraction step, as decreasing concentrations of organic solvents generally decreases the extraction efficiency of interfering substances such as plant pigments [23]. As shown previously, KAR_1_ and KAR_2_ are stable in both weakly acidic and neutral conditions (pH 5.0 and 7.0) [24]. Moreover, the use of acidic conditions at low temperature minimizes enzymatic activity during plant tissue extraction [28]. Similarly to SL extraction [27], the percentage of the organic component in the solvent was chosen to provide efficient solubility of KARs, as well as to meet compatibility with loading conditions of SPE sorbents used afterwards. Therefore, four polar solvents (10% methanol, 10% acetonitrile, 10% methanol acidified with 0.1% formic acid and 10% acetonitrile acidified with 0.1% formic acid) were tested as suitable solvents and the stability of KARs was evaluated (Additional file 1). The solvent mixtures were spiked with known amounts of KAR_1_ and KAR_2_, and then analysed by UHPLC–MS/MS. Recovery of each compound was calculated as its peak area relative to the corresponding peak’s area in control samples. The approximately twofold lower yield of KARs in acetonitrile compared to methanol probably indicates their different solubility. The results also showed lower concentrations of KAR_1_ and KAR_2_ quantified in spiked non-acidified solvents. Interestingly, the maximum recovery was not achieved with any of the solvents studied, which could also be due to losses during the evaporation step and/or the analyte adsorption to plastic or glass containers [29]. Moreover, Scaffidi et al. [30] showed ultraviolet-dependent degradation of KAR_1_ to head-to-head cage photodimers upon irradiation with a solar light source. It can be expected that KARs decay rapidly in natural sunlight [3]. However, these possible factors contributing to KAR losses were not investigated. In summary, the highest recovery was obtained with acidified 10% methanol (Additional file 1), therefore, this solution was used for extraction in all subsequent optimization procedures.

**Fig. 3:**
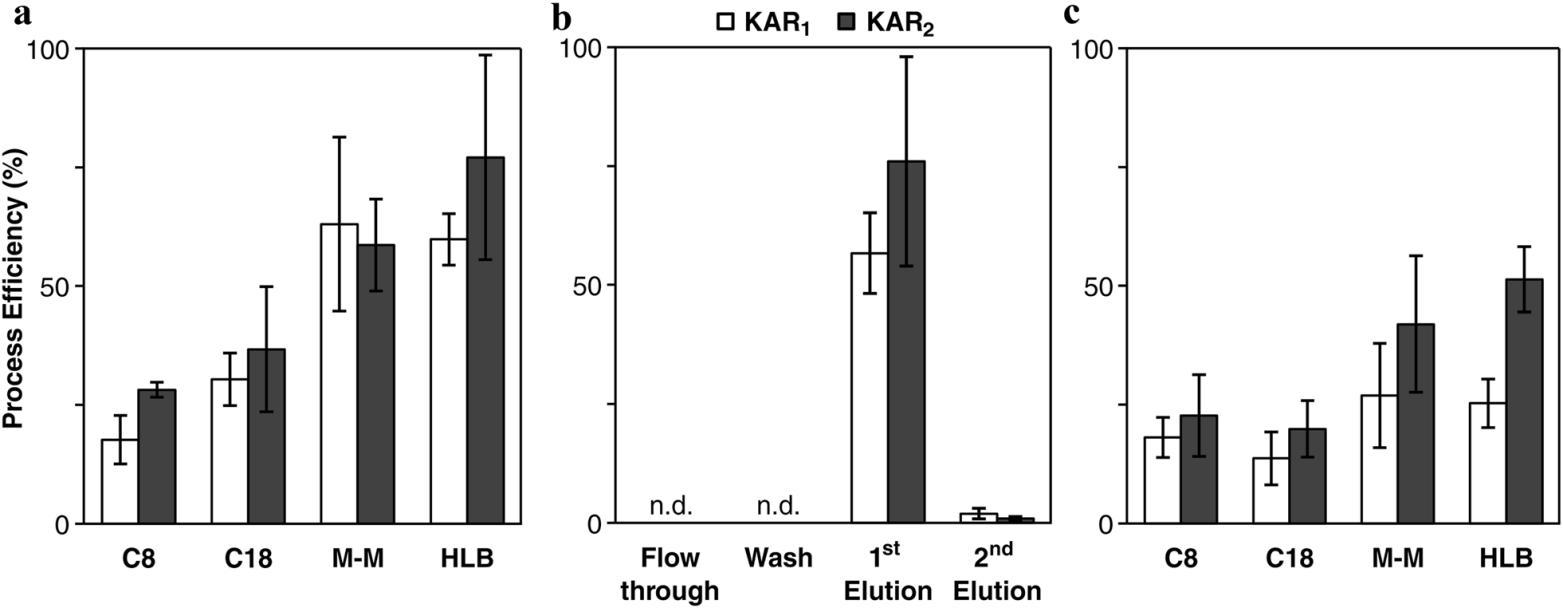
Process efficiency of the SPE-based method for KAR isolation. **a**, **c** Recovery (%) of KAR_1_ and KAR_2_ purified by four different SPE sorbents (C8, C18, Isolute M-M and Oasis HLB) without (a, 0 mg FW) and with plant matrix (c, 10 mg FW). **b** Representative test of loading capacity and extraction recoveries at different steps during the purification protocol described in Fig. 4. For all experiments, 10 pmol of each analyte was added to 1 ml of 10% methanol acidified with 0.1% formic acid without and with the presence of a plant tissue. The samples were then extracted and applied on SPE columns. After UHPLC–MS/MS analysis, the KARs' peak areas were compared to the peak’s areas of the original stock and expressed as a percentage recovery. Values are means ± SD (n = 3)

The next step in the method optimization was focused on the selection of SPE sorbents suitable for the subsequent pre-concentration of KARs from plant samples. As mentioned above, the combination of an optimized extraction protocol with a simple one-step purification allows the reduction of a complex plant matrix resulting in sensitivity and selectivity enhancements of the final MS-based analysis [13]. Moreover, structurally similar SLs can be isolated using reverse phase [31], polymeric [32] or ion-exchange sorbents [33]. To maximize the yield of the SPE step and to reduce the effect of the plant matrix, four different SPE sorbents based on reverse phase (RP) or multiple-mode interactions were tested. The overall process efficiency (PE) of each analyte was then compared (Fig. 3b). Due to their polar character (log P < 0), KARs were weakly retained on silica-based RP resins with short and long carbon alkyl chains (C8 and C18). The average recoveries of KARs in the elution fraction ranged from 18 to 28% and from 30 to 37% for C8 and C18, respectively. The use of ISOLUTE multimode sorbent combining of non-polar (C18), strong cation exchange (SO_3_^–^) and strong anion exchange (–NR_3_^+^) retention mechanisms showed higher recoveries of KAR_1_ and KAR_2_ (63 ± 18 and 59 ± 10%, respectively). Similarly, application of polymer-based RP columns with a hydrophilic-lipophilic-balance (HLB) water-wettable sorbent resulted in 60% yield of KAR_1_ and almost 80% yield of KAR_2_ (Fig. 3a). To determine losses during the purification process, we also monitored the loading capacity and extraction recovery in different steps of the tested protocol (flow through, wash and elution). For example, using the HLB resin, no KARs were eluted from the sorbents during sample application and washing steps (Fig. 3a). Under our experimental conditions, all tested KAR standards were mostly eluted with 2 ml of 80% methanol with average recovery 66%. The second elution step (2 ml 80% methanol) did not significantly increase the yield of KARs (Fig. 3b).

To evaluate the behaviour of sorbents in the presence of a complex matrix, a test of four sorbents was also performed with plant extract spiked with known amounts of KARs. As shown in Fig. 3c, the average recoveries of KARs were two-fold lower for C8 and C18 sorbents compared to two other SPE columns. Moreover, C8 and C18 resins do not efficiently remove interfering substances from complex biological matrices that can contaminate the MS instrument and interfere with column binding, elution and ionization [34]. Our findings showed similar KAR_1_ pre-concentration process efficiencies for ISOLUTE multimode and HLB sorbents, in average 34 and 38%, respectively. However, the difference is evident in the higher reproducibility of the results obtained using the Oasis HLB column (RSD% < 20; Fig. 3c). These findings were in good agreement with previously described SPE method for SLs determination [27]. Additionally, comparable results were achieved with only one-fifth amount of sorbent required to perform the clean-up procedure (150 mg vs. 30 mg of the Oasis HLB cartridges for SL vs. KAR isolations, respectively). Moreover, polymer-based SPE is widely used for extraction of plant hormones from minute samples due to higher stability and sample capacity [23, 34, 35].

**Fig. 4:**
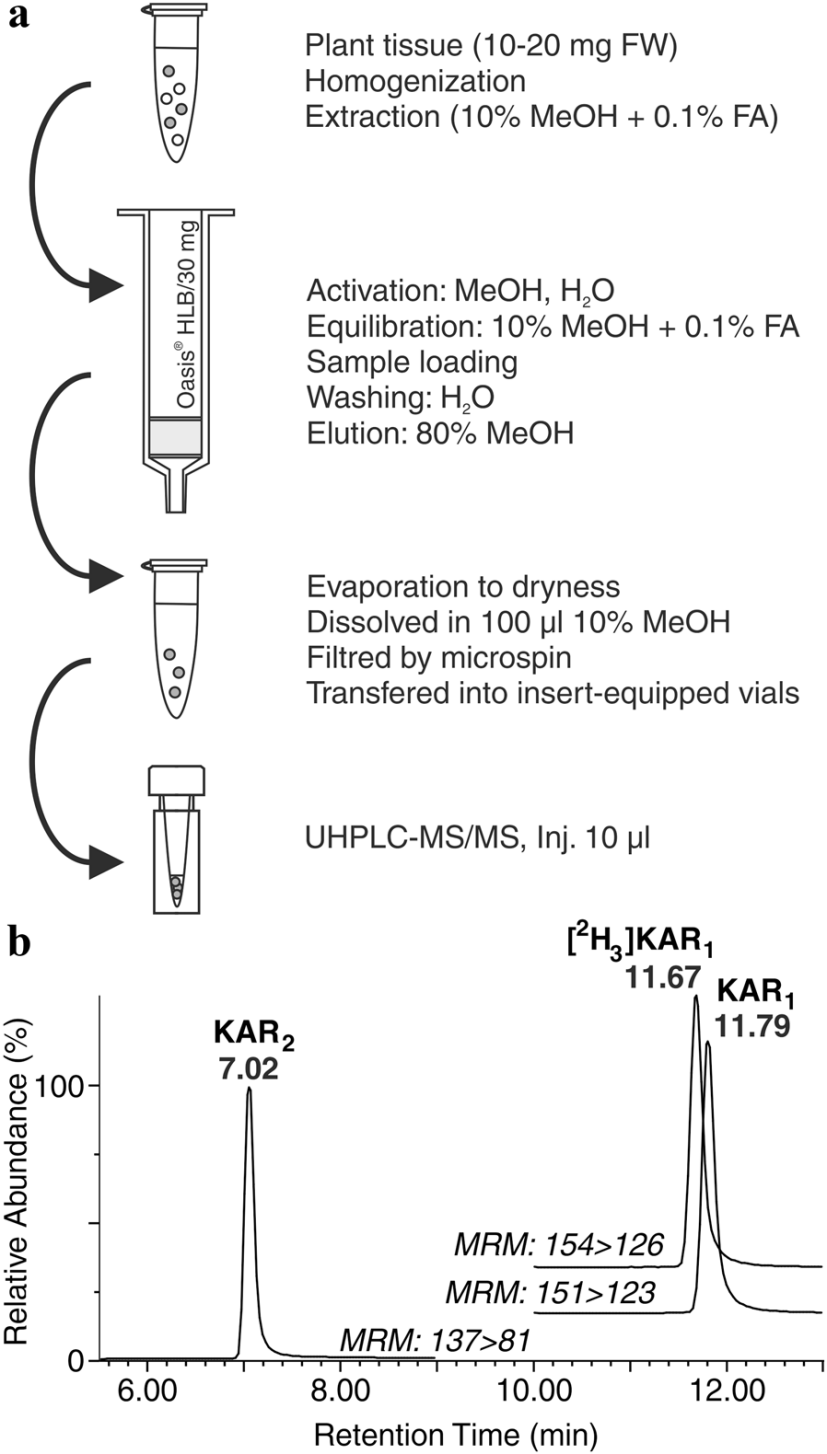
Optimized protocol for KAR determination in plant tissues. **a** Scheme of sample microextraction and purification using one-step solid phase extraction with Oasis HLB columns (30 mg/1 ml). **b** Representative multiple-reaction monitoring chromatograms of KAR_1_ and KAR_2_ with appropriate internal standard [^2^H_3_]KAR_1_ containing 1 pmol of each derivative per injection separated by optimized UHPLC–MS/MS method. FA – formic acid, MeOH – methanol, MRM – multi reaction monitoring

Based on the obtained data, we selected the Oasis HLB columns packed with 30 mg of *m*-divinylbenzene and *N*-vinylpyrrolidone copolymer for further characterization of the extraction protocol (Fig. 4a). As mentioned above, optimized extraction in acidified 10% methanol stabilized KAR metabolites and also reduced the concentrations of interfering compounds such as lipids and plant pigments. Moreover, one-step purification protocol including washing (water) and elution (80% methanol) steps pre-concentrated the KAR metabolites in the purified plant extracts. All steps together combine approaches suitable for KAR isolation before subsequent UHPLC–MS/MS analysis (Fig. 4b).

### Optimization of UHPLC–MS/MS method

Over the last decade, UHPLC has been the most commonly used rapid LC technique in bioanalysis, including methods described for various PGRs [23, 34–40]. In order to quantify KARs, a one-step purification method was combined with a fast chromatographic run based on sub-2-μm ethylene-bridged hybrid (BEH) polymer-based particles. Initially, purified plant tissue samples were separated on a short reversed-phase BEH column with a total run time of 7.0 min, including equilibration [24]. However, in the absence of adequate chromatography to separate the endogenous metabolites from the interfering compounds, the LC–MS/MS method will not be specific to the analyte of interest [41]. Due to the co-elution of [^2^H_3_]KAR_1_ with interfering substances originating from a complex multi-component plant matrix (Additional file 2), the previously published separation method had to be modified. Instead of a 5 cm-length column, a three times longer BEH Shield RP18 column with the same particle size (1.7 µm) and the same diameter (2.1 mm) was applied. In this case, extending the column length increased the number of theoretical plates and therefore improved the chromatographic resolution [42]. Thus, without changing the mobile phase composition (methanol and water acidified with 0.1% formic acid), the linear gradient was only slightly modified (see chromatographic parameters in the chapter UHPLC–MS/MS Conditions). As shown in Additional file 2, the use of a larger amount of BEH sorbent together with a two-fold extension of the chromatographic run provided better base-line separation from the sample background interferences. Moreover, there was no visible deterioration in the peak shape of [^2^H_3_]KAR_1_. Under our chromatographic conditions, KAR_2_ and KAR_1_ compounds were reproducibly eluted in 7.02 ± 0.03 min and 11.79 ± 0.04 min, respectively (Fig. 4b). Due to the chromatographic isotope effect [43], deuterated analogue was eluted slightly earlier than corresponding authentic standards at a constant time difference 0.12 min. Finally, to maximize the MS signal intensity for each compound, two time scan segments were used for analysis of KAR_2_ and KAR_1_ (5.5–9.0 and 10.0–13.0 min, respectively), see Fig. 4b.

The high selectivity of MS/MS instruments based on specific data acquisition modes, such as multiple reaction monitoring (MRM), allows precise quantification of trace analytes in complex matrices [12]. In accordance with the previously published method, karrikins were detected by a triple quadrupole mass spectrometer equipped with an electrospray ionization (ESI) in positive mode. All analytes, including the newly synthetized internal standard [^2^H_3_]KAR_1_, gave a strong signal from the protonated precursor [M + H]^+^ and two most abundant product ions [24]. Therefore, quantification and confirmation MRM transitions for each compound were selected and used to determine KARs under the optimized MS conditions listed in Additional file 3. As mentioned above, the MRM channels were time sectored to increase the cycle time for each analyte and acquired sufficient sensitivity (Fig. 4b). In addition, the automatically calculated dwell time provided at least 16 data points per chromatographic peak to ensure reliable integration [23]. Under these parameters, the limits of detection (LOD) and quantification (LOQ), defined as signal-to-noise ratios (3:1 and 10:1, respectively), were determined for each analyte. Similarly to the previous published study [24], the minimum detectable amounts of KAR_1_ and KAR_2_ were close to 0.1 fmol. To test the method linearity, a 14-point calibration curve (Cal 1) was constructed for each target analyte by plotting a known concentration of non-labelled analyte ranging from 0.25 fmol to 1000 pmol and a fixed amount of a deuterium labelled IS (0.5 pmol of [^2^H_3_]KAR_1_). The curves had a linear range spanning at least 4 orders of magnitude from 0.01 to 25 pmol with a coefficient of determination *R*^*2*^ ≥ 0.999 (Additional file 4). The optimized analytical method (isolation and quantification) was validated to further allow the analysis of KAR concentrations in plant tissues.

### Method validation

The developed method combines a convenient sample purification process based on one-step SPE with UHPLC–MS/MS and enables precise quantification by stable isotope dilution method (SIDM). As shown in Fig. 3c, the pre-concentration process efficiency of KAR_2_ was almost two-fold higher in the presence of *Arabidopsis* matrix compared to the yield of KAR_1_. These results indicated the future problems in the application of solvent-only calibration (Cal 1) for the determination of KAR_2_ based on the SIDM. This difficulty could be solved by the second stable isotope labelled IS for KAR_2_ or by spiking target analytes into biological matrix spanning the intended calibration range [44]. Therefore, the unavailability of deuterium labelled KAR_2_ was replaced by adding known concentrations of the reference standard into a qualified batch of sample matrix. Every effort was made to prepare the standard calibrators in a biological matrix, which matched the chemical background with respect to species, composition, and sample pre-processing [45]. Two matrix-matched calibration sets (Cal 2 and 3) were prepared for each KAR analyte and further investigated. Similar to Cal 1, the calibration solutions contained various concentrations of each unlabelled KAR metabolite and a defined concentration of the stable isotope labelled IS. The first calibration series (Cal 2) was dissolved in plant matrix samples obtained after the SPE step (10 mg FW of *Arabidopsis* seedlings pre-extracted in acidified 10% methanol and pre-purified on an HLB column). The second matrix calibration curve (Cal 3) was constructed using KAR standards added to the crude *Arabidopsis* extract and then purified by SPE (Fig. 4a). Following regulatory guidelines on bioanalytical method validation [18], all calibration standards at six concentration levels were analysed in duplicate to generate a linear calibration curve. Both matrix calibrations showed a linear range extending from 0.05 to 10 pmol with *R*^*2*^ ≥ 0.999 (Additional file 4).

**Fig. 5:**
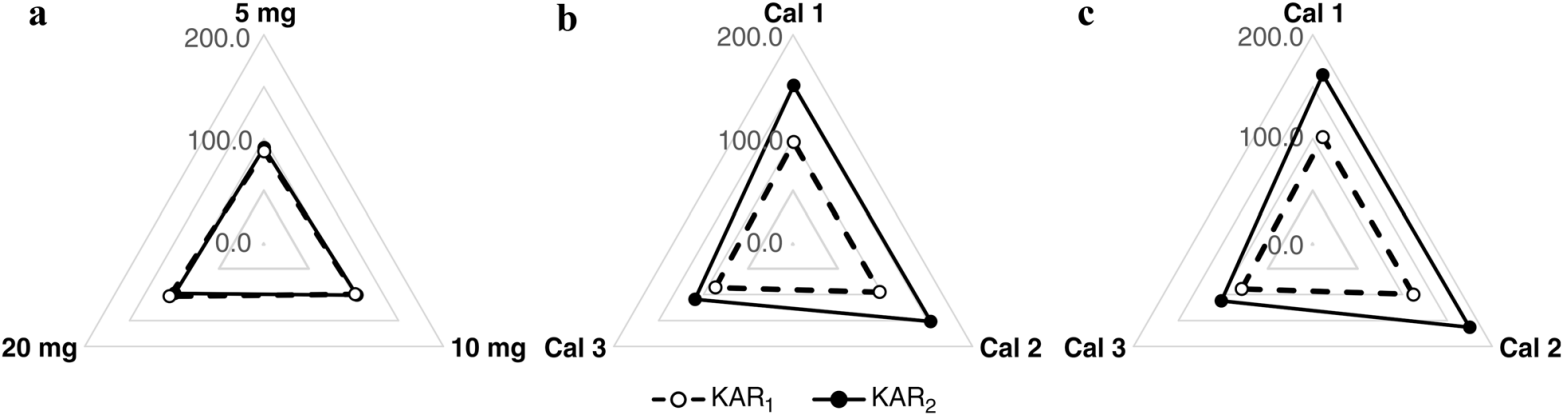
Validation of karrikin quantification method using the parameters of recovery (**a**), matrix effect (**b**) and process efficiency (**c**). The recovery (%) was calculated from the peak area of each compound (10 pmol of KAR_1_ and KAR_2_) added to the plant extract (5, 10 and 20 mg FW of ten-day-old *Arabidopsis* seedlings). Matrix effect (%) and process efficiency (%) were calculated with solvent-only calibration in methanol (Cal 1), calibration dissolved in the plant matrix blanks obtained after the SPE step (Cal 2), and matrix-matched calibration prepared similarly to the sample according to developed purification protocol (Cal 3)

Hereafter for method validation shown in Fig. 5 and Additional files 5, 6, the parameters of recovery (RE), matrix effect (ME) and process efficiency (PE) were determined using three sets of samples spiked with 10 pmol of KAR_1_ and KAR_2_ as described in the chapter Method development and validation. First, we compared the absolute peak areas obtained for neat solution standards with the corresponding peak areas for standards spiked into plant extracts after purification into plant extracts and peak areas for standards spiked before the SPE step [46]. Moreover, the retention capacity of the Oasis HLB sorbent was also tested with increasing amounts of plant matrix (5, 10 and 20 mg FW of *Arabidopsis* seedlings). In general, REs express the proportion of analytes obtained from the sample during its purification by SPE [47]. Surprisingly, the recovery of KAR metabolites was not influenced by higher sample weights, and the use of 30 mg cartridges was sufficient to maximize the yield of the SPE step (Fig. 5a). On the other hand, the negative effect of the sample matrix was evident from the values of ME and PE, reaching on average only 30 and 50% for KAR_1_ and KAR_2_, respectively (Additional file 5). Our results showed a combined effect of possible losses during sample preparation and suppression of analyte ionization in the ion source by co-eluting compounds originating from the sample matrix.

In an additional step, we determined the concentration of each analyte in all enriched samples using the ratio of the analyte signal to the internal standard signal and the corresponding calibration curve (Additional file 4). Solvent-only calibration (Cal 1) and two matrix-matched calibration series (Cal 2 and Cal 3) were compared to demonstrate the equivalence of qualitative results. The KAR concentrations in *Arabidopsis* samples were then used to calculate IS-normalized ME and PE values [48], see chapter Method development and validation. Overall, the IS-normalized values of KAR_1_ determined by the Cal 1–3 curves were quite similar, ranging from 87 to 98% for ME and from 90 to 102% for PE. Both values confirmed that the use of [^2^H_3_]KAR_1_ made it possible to correct for the ME and PE observed for the target analyte. Conversely, the results of IS-normalized ME and PE for KAR_2_ indicated incorrect quantification applying the Cal 1 and Cal 2 curves (Fig. 5b, c). The calculated values of ME and PE were in the range 139% to 153% and 141% to 164%, respectively (Additional file 6). The use of the SIDM in combination with the Cal 3 calibration achieved a successfully valid quantification of KAR_2_ (Fig. 5b, c). On average, the IS-normalized ME was only 112% and the IS-normalized PE was 114% (see Additional file 6). Our findings confirmed the need for standard solutions prepared in plant extracts similar to that of the sample [18]. The use of Cal 3 can compensate for most of the errors obtained during the whole procedure (Fig. 4).

Finally, the effectiveness of the presented method was demonstrated by measurement of spiked samples of ten-day-old *Arabidopsis* (10 mg FW) with a standard mixture containing 1, 5 and 10 pmol of authentic KAR standards. The newly synthesized isotopically labelled IS (10 pmol of [^2^H_3_]KAR_1_) was also added to apply the SIDM approach. After extraction in acidified 10% methanol and subsequent SPE purification, the samples were analysed by optimized UHPLC–MS/MS method (Fig. 4). The concentration of both KARs was determined using three different calibration series (Cal 1, Cal 2 and Cal 3) and the methods’ precision and accuracy were calculated (Table 1). Similar to the results of ME and PE assays, the final validation experiment indicates the requirement for a matrix-matched calibration passed through the SPE cartridge. The method’s precision was quantified by evaluating the closeness of a series of replicate samples, and was expressed in terms of the relative standard deviation (RSD%). The RSD% were below 5% for all tested levels of both KAR compounds (see Table 1). The accuracy of the analytical method, defined as the difference between the levels obtained in an analytical run and the accepted reference value, was estimated by percentage bias (%Bias). In general, analysis of KAR_1_ was accurate applying the Cal 1 and Cal 3 calibration series (bias below 27% and 13%, respectively). The accuracy of KAR_2_ quantification was insufficient when the matrix-free calibration (Cal 1) was applied. The acquired data showed that the use of matrix calibrators improved the method accuracy, however, a combination of the stable isotope dilution method with a matrix-matched calibration prepared similarly to the sample (Cal 3) was only applicable (Table 1). The accuracy means for KAR_2_ were 132.0%, 58.5% and –3.7% for the Cal 1, Cal 2 and Cal 3 curves, respectively. Hence, accuracy of the developed analytical approach is satisfactory for the detection of trace components within ± 15% of the true amounts in a complex plant matrix [49]. All validation parameters of the developed method were comparable to the results reported by authors using LC–MS/MS for plant hormones analysis in plant tissue samples [23, 34, 35, 38–40, 50]. In conclusion, applying the Cal 3 calibration, the precision and accuracy demonstrate the methods’ reliability and usefulness for routine KAR analysis in plant material.

**Table 1:** Method validation

Compound	Calibration curve	Determinated spiked KARs content [pmol]			Method precision [RSD%]			Method accuracy [%bias]		
		1	5	10	1	5	10	1	5	10
KAR_1_	Cal 1	1.27 ± 0.03	5.60 ± 0.05	11.09 ± 0.49	2.4	1.0	4.5	27.1	12.0	10.9
	Cal 2	1.58 ± 0.04	6.89 ± 0.07	13.57 ± 0.60	2.4	1.0	4.4	58.0	37.7	35.7
	Cal 3	1.08 ± 0.03	5.39 ± 0.06	11.29 ± 0.54	2.6	1.0	4.8	8.2	7.7	12.9
KAR_2_	Cal 1	2.79 ± 0.12	10.50 ± 0.28	20.72 ± 0.74	4.2	2.6	3.6	178.8	110.1	107.2
	Cal 2	1.72 ± 0.08	7.39 ± 0.21	15.61 ± 0.61	4.6	2.9	3.9	71.5	47.8	56.1
	Cal 3	1.06 ± 0.05	4.47 ± 0.13	9.34 ± 0.36	4.5	2.9	3.9	6.0	− 10.6	− 6.6

Analytical precision (RSD%) and accuracy (%bias) of whole procedure shown for different amounts of karrikins (1, 5 and 10 pmol). The extract of 10 mg (FW) Arabidopsis sample was spiked from 1 to 10 pmol of authentic KAR standards, purified by SPE and analysed by UHPLC-MS/MS. Concentrations of KARs were quantified using the standard isotope dilution method combined with calibration curves without (Cal 1) and with (Cal 2 and 3) plant matrix. Values are means ± SD (n = 3)

### Measurement of karrikin concentrations in plants

It is one of a fundamental biological interests to improve our knowledge about how small signalling molecules, such as karrikins, regulates vital processes in plants. The study of KARs’ mode of action should include not only the signalling pathways, transcription factors and responsive genes, but also knowledge of their concentration levels in various plant organs. The use of modern analytical tools allows accurate detection and quantification of low abundant compounds.

To assess the applicability of the newly developed approach, we quantified KAR levels in ten-day-old *Arabidopsis* seedlings (10 mg FW) grown on media supplemented with different concentrations of KAR compounds (Table 2). After extraction in acidified 10% methanol and subsequent SPE purification, the samples were analysed by optimized UHPLC–MS/MS method (Fig. 4). Units and tens pmol/g FW of KAR_1_ and KAR_2_ were detected in samples grown on medium enriched with a mixture of pure karrikin standards at 100 nM and 1 µM concentrations, respectively. Interestingly, elevated karrikin levels were determined in *Arabidopsis* samples treated with the highest concentration (10 µM of each compound). Our results suggest that this approach enables the targeted and sensitive determination of karrikin levels and thus allows the detailed study of the physiological roles and modes of action of KAR in plants. Interestingly, our findings indicate a different accumulation rate of KARs in plant tissue after treatment with their low or high concentrations, however, the mechanism of this process is still unknown. This effect could be further investigated in further attempts to modify plant development by exogenously applied karrikins in order to improve crop yields [51]. In addition, our quantitative data on KAR levels in plant tissues cannot be directly compared with previous reports, since KARs have been only quantified in smoke water samples so far [6, 24].

**Table 2:** Karrikin quantification in ten-day-old *Arabidopsis thaliana* seedlings grown on media supplemented with KAR_1_ and KAR_2_

Concetration of KARs in growing media [µmol/l]	KAR content [pmol/g FW]	
	KAR_1_	KAR_2_
0.1	1.8 ± 0.2	5.5 ± 0. 4
1	60.9 ± 8.9	66.1 ± 11.4
10	1577.9 ± 111.4	1065.9 ± 101.5

Values are means ± SD (n = 3)

## Conclusions

Precise measurements of karrikins are technically highly challenging and seed-germination bioassays are mainly used to detect activity [3]. In the presented study, we have developed a new sensitive and specific method for isolation and analysis of karrikin compounds in small amounts of plant tissue samples. The protocol is based on a solid phase extraction combined with a sensitive UHPLC–MS/MS method. Quantification of the analytes was performed by a stable isotope dilution method employing a newly synthetized isotopically labelled internal standard. This new method was fully validated and successfully applied for KAR analysis in treated *Arabidopsis* samples. Our results demonstrate the applicability of the developed methodology for routine analyses and for monitoring KARs in complex biological matrices. Based on the synthesis of new standards, potential applications of this approach in analyses of all described KARs in one-step SPE/UHPLC–MS/MS runs are under developed. We are also aware that employment of novel atmospheric pressure ionization interfaces can also lead to further improvements in our quantitative method.

## Methods

### Reagents and materials

Methanol (gradient grade for liquid chromatography), acetonitrile (gradient grade for liquid chromatography) and water (for chromatography) were obtained from Merck (Darmstadt, Germany). Oasis® HLB (RP, polymer-based SPE cartridges, 30 mg/1 ml) were purchased from Waters (Milford, MA, USA), Isolute® M-M (100 mg/1 ml) from Biotage (Uppsala, Sweden), Bond Elut-C8 (500 mg/3 ml) from Agilent Technologies (Santa Clara, CA, USA), and Spe-ed SPE C18 (100 mg/1 ml) from Applied Separations (Allentown, PA, USA). The methylboronic acid (methyl-d3) was obtained from Cambridge Isotope Laboratories Inc. (Tewksbury, MA, USA) Formic acid and other reagents for chemical synthesis were purchased from Sigma-Aldrich (St. Louis, MI, USA). KAR_1_ and KAR_2_ were synthesized as described previously by Hrdlička et al. (2019) [24]. The solid substances of authentic KAR standards were dissolved in methanol to a concentration 10^–3^ mol/l and then gradually diluted to lower concentrations.

### Biological material

*Arabidopsis thaliana* (ecotype Col-0) seedlings were grown on full MS medium with 1% sacharose and 1% agar (Duchefa Biochemie, Haarlem, Netherlands) at pH 5.7 in a growth chamber under long-day conditions at 23 °C under a 16-h photoperiod. Stock solution of karrikin compounds (KAR_1_ and KAR_2_) was dissolved in deionized water and applied to cultivation media at the final concentration 0.1, 1 and 10 µmol/l. The ten-day-old plants were harvested, carefully rinsed in distilled water (three times) and subsequently dried with filter paper to avoid contamination of plant surface. The samples were immediately plunged into liquid nitrogen, weighed and stored at -80 °C until extraction and purification before analysis. Untreated *Arabidopsis* plants were grown under the same conditions as described above and used for method development and validation or as controls for KAR quantification.

### Synthesis of isotope labelled standard

The mixture of palladium diacetate (16.0 mg, 72.5 µmol), S-Phos (2-dicyclohexylphosphino-2′,6′-dimethoxybiphenyl) (90.0 mg, 0.218 mmol) and potassium phosphate (70.0 mg, 0.60 mmol) in toluene (3.0 ml) was stirred at room temperature in a sealed tube for 30 min under an argon atmosphere. The solution of 3-bromo-furo[2,3-c]pyran-2-one (63.0 mg, 0.29 mmol) and methylboronic acid (methyl-d3) (25.0 mg, 0.44 mmol) in toluene (2.0 ml) was added and to the reaction mixture was heated at 100 °C with stirring for 48 h in a sealed tube under an argon atmosphere. After cooling to room temperature, the reaction mixture was filtered through celite and washed with toluene (15.0 ml). The filtrate was then evaporated under reduced pressure and the residue was suspended in dichloromethane (20.0 ml). The organic phase was washed with water, brine, dried over anhydrous sodium sulphate and evaporated under reduced pressure. The crude product was purified by column chromatography on silica using mobile phase petroluem ether – ethylacetate – triethylamine (3:1:0.025) and subsquently by column chromatography on silica using mobile phase chloroform-triethylamine (97.5:2.5) and Merck silica gel Kieselgel 60 (230–400 mesh). Yield: 22 mg (49%).

The GC–MS analyses were performed on GC–MS QP2010 Ultra (Shimadzu, Kyoto, Japan). The separation was performed on a Zebron ZB-5MS capillary column, length: 30 m, inner diameter 0.32 mm, thin layer 0.25 µm. Helium was used as a carrier gas at constant flow 1.20 mL.min-1. The injection volume of the samples was 1 µl, sample concentration 1.0 µg.mL-1 in splitless mode. The injector temperature was 260 °C, sampling time 1 min, solvent cut time was 1.5 min. The temperature programme started at 60 °C held for 1 min and was followed by temperature rate 20 °C.min-1 to 280 °C which was held for 5 min. The interface temperature was 280 °C. The ion source operated with collision energy 70 eV at temperature 250 °C and detector voltage 0,7 kV. The mass spectra were scanned in a range 50–650 m/z in a speed 2000 scans.s-1. GC–MS (EI, 70 eV): retention time 8,27 min; *m/z* (rel.int.): 123.10 (100), 153.05 (69), 96.10 (26.5), 68.10 (24.5), 97.1 (16.5).

The analysis of HPLC–PDA-(ESI +)MS purity was performed on an Acquity UPLC® H-Class System combined with Acquity PDA and Acquity QDa detectors (all from Waters) with detection at wavelengths of 210–400 nm and electrospray ionization in positive mode in *m/z* 50–1000 range, respectively. Briefly, 10 μl of the IS (concetration 10 μg/ml) was injected onto a thermostated (25 °C) RP column (150 mm × 2.1 mm, 5 μm C18 Symmetry, Waters) and eluted at a flow rate of 0.3 ml/min using a linear gradient of 15 mM ammonium formate at pH 4.0 (A) and pure methanol (B) as follow: 0 min, 10% B; 0–25 min, 10–90% B, 25–35 min, 90% B. The column was then re-equilibrated under the initial conditions (10% B) for 10 min. The MS conditions were set for source/probe temperatures at 120/600 °C and capillary/cone voltages of + 800/ + 15 V. Nitrogen was used the desolvation gas. HPLC–DAD–(ESI +)MS: *m/z* 154.1 (purity 96.2%).

^1^H and ^13^C-NMR spectra were recorded on a Jeol 500 ECA instrument operating at 500 MHz for 1H and 126 MHz for 13C. Chemical shifts are reported in ppm. Coupling constants (J) are reported in Hertz (Hz), and the following abbreviations are used: singlet (s), doublet (d). ^1^H NMR (CDCl3): 6.509 (d, J = 5.50 Hz, 1H, CH), 7.322 (d, J = 5.50 Hz, 1H, CH); ^13^C NMR (CDCl3): 7.58, 101.30, 105.00, 128.64, 141.75, 143.28, 150.41, 172.38.

### Extraction and purification optimization

All samples were homogenized and weighed under liquid nitrogen into 2 ml plastic microtubes (Eppendorf, Germany) containing three 2 mm ceria-stabilized zirconium oxide beads. 3-(^2^H_3_)methyl-*2H*-furo[2,3-c]pyran-2-one ([^2^H_3_]-KAR1, 10 pmol) was used as internal standard to check the recovery during purification and to validate the determination of KAR_1_ and KAR_2_. The frozen plant material (5–20 mg FW) was extracted in 1 ml of ice-cold extraction solution (0.1% formic acid in 10% methanol, v/v) using vibration mill MM 301 (27 Hz, 3 min; Retsch GmbH & Co. KG, Haan, Germany). Samples were sonicated (4 °C, 3 min; Elma, Germany) and subsequently incubated using a benchtop laboratory rotator Stuart SB3 (4 °C, 30 min; Bibby Scientific Ltd., Staffordshire, UK). After centrifugation (14,000 rpm, 15 min; Beckman Coulter, Brea, CA, USA), the supernatants were purified by RP polymer-based solid phase extraction Oasis® HLB columns (1 cc per 30 mg, Waters). The SPE sorbent was activated sequentially by 1 ml of 100% methanol and 1 ml of deionized water, then equilibrated with 1 ml extraction solution (0.1% formic acid in 10% methanol, v/v). After sample loading, the HLB column was washed with 1 ml of deionized water and analytes were eluted with 2 ml of 80% methanol (v/v). The eluted samples were evaporated to dryness at 30 °C under a stream of nitrogen (TurboVap LV, Biotage) and stored at -20 °C until UHPLC-MS/MS analysis.

### Method development and validation

The stability of KARs was tested in triplicate by adding 10 pmol of KAR_1_ and KAR_2_ to 1 ml of 10% methanol or 10% acetonitrile (non-acidified and/or acidified with 0.1% formic acid). The samples were thoroughly mixed, evaporated to dryness under a stream of nitrogen, re-suspended in 100 μl of 10% MeOH and then analysed by UHLC-MS/MS method (10 μl per injection). Finally, recoveries of each compound (percentages of average peak areas in each solvent relative to respective peak areas obtained from analyses of reference samples) were calculated (Fig. 3).

To develop an isolation protocol, four SPE sorbents (Bond Elut-C8, Spe-ed C18, Isolute M-M and Oasis HLB) were tested using a mixture of 10 pmol of KAR_1_ and KAR_2_ without (0 mg FW) and/or with plant matrix. Briefly, 10 mg FW of Arabidopsis seedlings was extracted in ice-cold acidified 10% methanol spiked with known amounts of KARs. All tested sorbents were activated with 1 ml of 100% methanol and 1 ml of deionized water, and equilibrated with 1 ml of acidified 10% methanol (Fig. 4a). After samples loading (1 ml of plant extract or neat standard), each sorbent was washed with 1 ml of deionized water and analytes were then eluted by two-step elution using 2 × 1 ml of 80% methanol. The samples thus prepared were evaporated to dryness under a stream of nitrogen, re-suspended in 100 μl of 10% MeOH and then analysed by UHLC-MS/MS method (10 μl per injection). The performance efficiency of four different cartridges was calculated as percentages of average peak areas relative to the corresponding peak areas in control samples. All experiments were performed in triplicates.

For the method validation, three different calibration series (Cal 1, 2 and 3) were used. Solvent-only calibration curve (Cal 1) was constructed using serial dilutions of authentic standards and known concentrations of internal labelled standards in methanol. Furthermore, two matrix-matched calibrations, Cal 2 and Cal 3, were prepared using 10 mg FW of *Arabidopsis* seedling per calibration point. For calibration 2, KAR standards were dissolved in the plant matrix blanks obtained after the SPE step. Calibration curve 3 was constructed using plant extract spiked with a known amount of KARs purified by developed purification protocol. All calibration curves were analysed in duplicate and constructed using least square linear regression analysis method (Additional file 4).

To validate the isolation protocol (Fig. 4), three sets of samples were prepared in triplicate and analysed by the UHPLC–MS/MS system. In the first set, *Arabidopsis* seedlings (5, 10 and 20 mg, FW) were extracted by acidified 10% methanol spiked with 10 pmol of KAR_1_ and KAR_2_ and stable isotope labelled IS (10 pmol of [^2^H_3_]KAR_1_), and subsequently purified by the SPE protocol. In the second set, the same plant extracts passed through SPE sorbent and then were spiked with the analytes and IS (10 pmol of each compound). Third set consisted non-matrix samples representing a standard mixture (10 pmol of authentic compounds and IS) purified by the SPE step without plant extract. Non-normalized recovery (in percentages) was calculated as a ratio of average peak areas of a non-labelled analytes spiked before and after SPE purification [46]. Non-normalized matrix effect and process efficiency of the method were then expressed as the ratio of average peak areas of KARs spiked before and after extraction to average peak area of the same analyte standards, respectively (Additional file 5). Futhermore, IS-normalized PE a ME were calculated as a concentration ratio of Set1 and Set 2 to Set 3, respectively [48].

Finally, 10 mg FW of *Arabidopsis* seedlings was extracted in ice-cold acidified 10% methanol spiked with 1, 5 and 10 pmol of KARs and 10 pmol of stable isotope-labelled IS, and subsequently purified by the SPE protocol (Fig. 4). Concentrations of karrikins were quantified by UHPLC-MS/MS method using the standard isotope dilution method [52] in combination with three calibration series (Cal 1, Cal 2 and Cal 3). The precision of the method was expressed as the relative standard deviation (RSD%) of three replicate measurements. The method accuracy was expressed as a relative bias of the determined analyte concentrations compared with the spiked amounts of KAR standards (Table 2). All experiments were done in triplicates.

### UHPLC–MS/MS conditions

Karrikins were analysed by an Acquity UPLC® I-Class System combined with a Xevo™ TQ-XS triple quadrupole mass spectrometer (Waters). The samples were dissolved in 100 μL of 10% methanol (v/v), filtered using modified nylon 0.2-μm Centrifugal Filters and then transferred to insert-equipped vials. 10 μl of each sample was injected onto an Acquity UPLC® BEH C18 reversed-phase column (1.7 µm, 2.1 × 50 mm) and/or Acquity UPLC® BEH Shield RP18 column (1.7 µm, 2.1 × 150 mm). The column temperatures were held at 40 °C. The compounds of interest were separated by a 5-min gradient elution with the flow 0.4 ml/min using acidified methanol (A, 0.1% formic acid in methanol) and acidified water (B, 0.1% formic acid in water), as follow 0–1 min isocratic elution by 5% A, 1–3 min linear gradient to 20% A and 3–5 min isocratic elution by 20% A. After this, column was washed with 100% A for 1 min and re-equilibrated to the initial conditions (5% A) for 1 min. Using a 150 mm-length column, the modified gradient included a flow 0.2 ml/min and an extension of the linear gradient from 5 to 20% A in 1–6 min and an isocratic elution by 20% A in 6–10 min, followed by washing step with 100% methanol for 1 min and re-equilibration to initial conditions (2 min).

During the UHPLC-MS/MS acquisition, the effluent was introduced into the electrospray ion source of a triple quadrupole mass spectrometer operating in positive mode under the following conditions: capillary voltage, 0.5 kV; source/desolvation temperature, 120 °C/600 °C; cone/desolvation gas flow, 150/1,000 l/h; collision gas flow (argon), 0.15 ml/min. Karrikins were quantified in MRM mode using dwell time in automatic mode for 16 scan points per peak and optimized MS conditions (Additional file 3). Acquired data were processed by MassLynx™ MS Software with TargetLynx™ program (version 4.2, Waters, Milford, MA, USA).

## Supplementary Information

**Additional file 1.** KARs stability in different extraction solutions.

**Additional file 2.** Optimization of chromatographic separation of an isotope labelled standard.

**Additional file 3.** Optimized parameters for the quantification of karrikins by UHPLC-MS/MS.

**Additional file 4.** Calibration curves used to validate the method.

**Additional file 5.** Non-normalized recovery, matrix effect and process efficiency.

**Additional file 6.** Internal standard-normalized recovery, matrix effect and process efficiency.

## Data Availability

All relevant data can be found within the manuscript and its additional files.
