# Supplementary material for: A stable isotope dilution method for a highly accurate analysis of karrikins
Source: Plant Methods. 2021 Apr 1;17:37. doi: 10.1186/s13007-021-00738-1 (PMC8017846; doi:10.1186/s13007-021-00738-1)
Supplement: Supplementary file 1 — Additional file 1. KARs stability in different extraction solutions. [file 13007_2021_738_MOESM1_ESM.pdf]

Additional files

Additional file 1. KARs stability in different extraction solutions. The mixture of KAR standards (10pmol of KAR_1 and KAR_2) was dissolved in four different non-acidified and acidified polar solvents, evaporated and analysed by LC-MS/MS. Compound peak areas in each solvent were compared to the original reference samples and expressed as a percentage recovery. Values are means ± SD (n = 3).

Extraction solution	Recovery [%]	
	**KAR_1**	**KAR_2**
10% MeOH	35 ± 8	43 ± 9
10% MeOH + 0.1 % FA	60 ± 3	73 ± 4
10% ACN	23 ± 3	33 ± 3
10% ACN + 0.1 % FA	50 ± 3	64 ± 2
