# Supplementary material for: A stable isotope dilution method for a highly accurate analysis of karrikins
Source: Plant Methods. 2021 Apr 1;17:37. doi: 10.1186/s13007-021-00738-1 (PMC8017846; doi:10.1186/s13007-021-00738-1)
Supplement: Supplementary file 2 — Additional file 2. Optimization of chromatographic separation of an isotope labelled standard. [file 13007_2021_738_MOESM2_ESM.pdf]

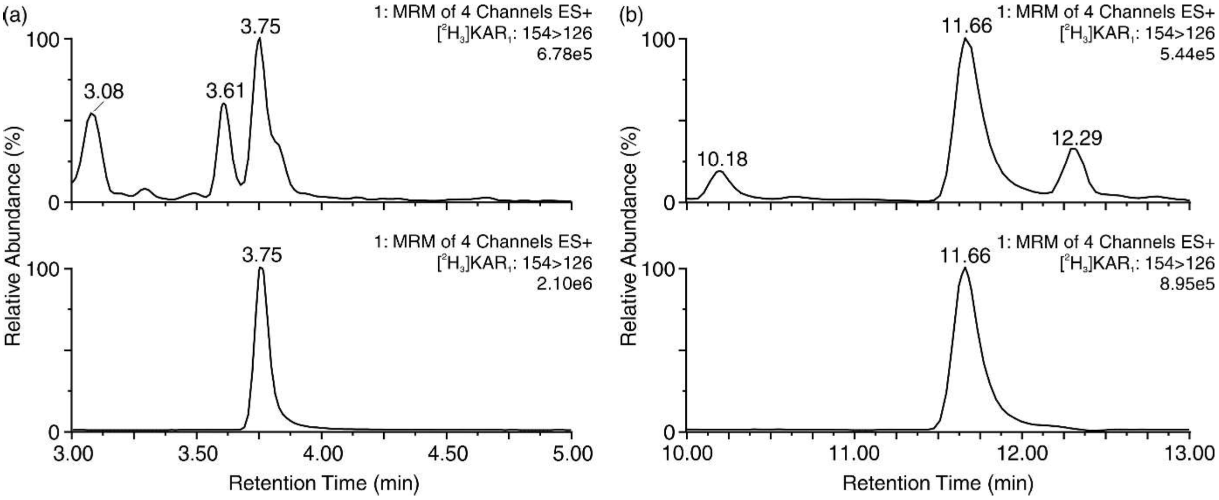

Additional file 2. Optimization of chromatographic separation of an isotope labelled standard. [$^{2}H_{3}$]KAR_{1} was separated onto (a) Acquity UPLC BEH C18 column (1.7 µm, 2.1 x 50 mm) and (b) Acquity UPLC BEH Shield RP18 column (1.7 µm, 2.1 x 150 mm) using short and long gradients, respectively. The retention times of [$^{2}H_{3}$]KAR_{1} added to plant extracts (10 mg FW) purified by SPE and determined by UPLC–MS / MS method (top) were compared to appropriate standard injected (bottom).
