# Supplementary material for: A stable isotope dilution method for a highly accurate analysis of karrikins
Source: Plant Methods. 2021 Apr 1;17:37. doi: 10.1186/s13007-021-00738-1 (PMC8017846; doi:10.1186/s13007-021-00738-1)
Supplement: Supplementary file 3 — Additional file 3. Optimized parameters for the quantification of karrikins by UHPLC-MS/MS. [file 13007_2021_738_MOESM3_ESM.pdf]

Additional file 3. Optimized parameters for the quantification of karrikins by UHPLC-MS/MS using an electrospray ionization (ESI) in positive mode. MRM, multiple reaction monitoring; n.c., not calculated.

	**KAR_1**	**KAR_2**	**[$^2H_3$]KAR_1**
Retention time (min)[a]	11.79 ± 0.04	7.02 ± 0.03	11.64 ± 0.04
MRM transitions (Quantitation / Confirmation)	151 > 123 / 151 > 67	137 > 81 / 137 > 109	154 > 128 / 154 > 70
Cone voltage (V)	25	20	20
Collision energy (eV)	18 / 22	20 /20	18 / 18
LOD (fmol)[b]	0.1	0.1	n.c.

[a] Means ± SD (n = 10).

[b] The signal-to-noise ratio was set to 3:1.
