# Supplementary material for: A stable isotope dilution method for a highly accurate analysis of karrikins
Source: Plant Methods. 2021 Apr 1;17:37. doi: 10.1186/s13007-021-00738-1 (PMC8017846; doi:10.1186/s13007-021-00738-1)
Supplement: Supplementary file 4 — Additional file 4. Calibration curves used to validate the method. [file 13007_2021_738_MOESM4_ESM.pdf]

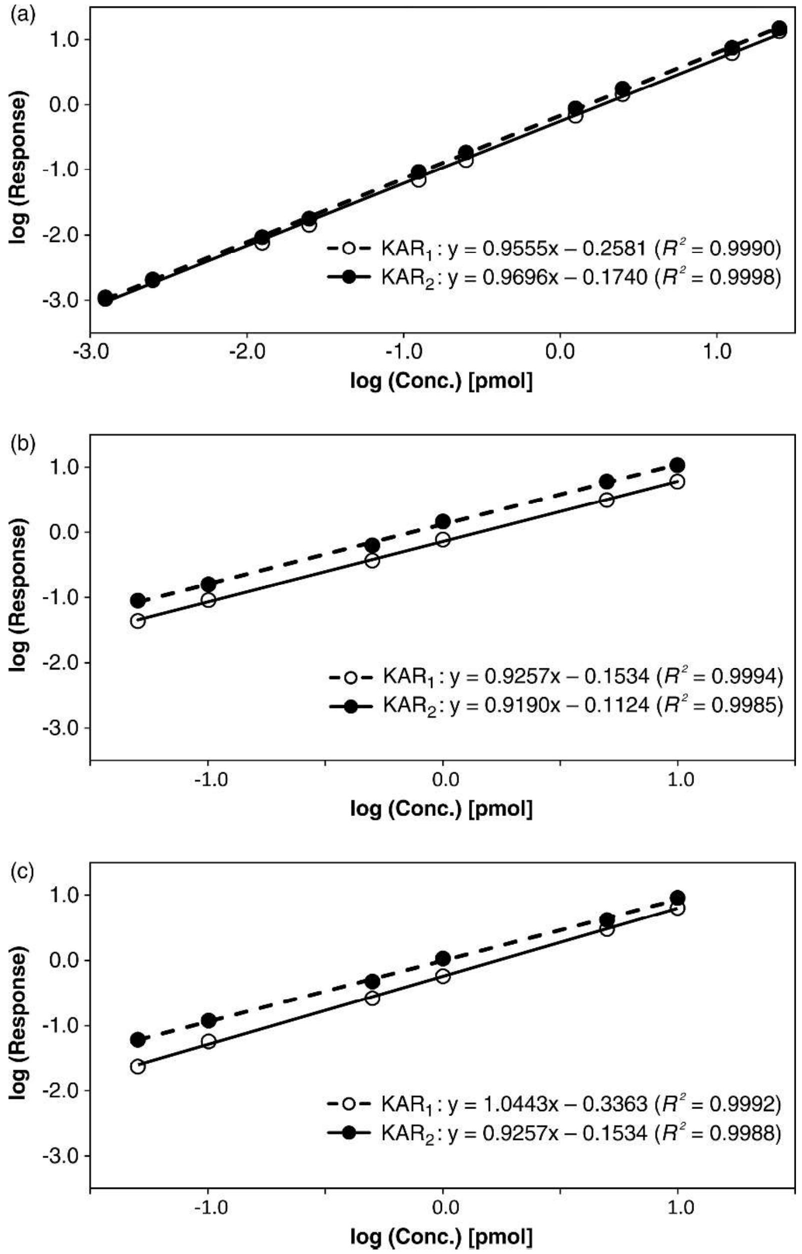

Additional file 4. Calibration curves used to validate the method. (a) Cal 1 – Solvent-only calibration in 10% methanol, (b) Cal 2 – calibration dissolved in the plant matrix blanks obtained after the SPE step, and (c) Cal 3 – matrix-matched calibration prepared similarly to the sample according to developed purification protocol. Each calibration curve is characterized by a regression equation and a coefficient of determination (R^2).
