# Supplementary material for: A stable isotope dilution method for a highly accurate analysis of karrikins
Source: Plant Methods. 2021 Apr 1;17:37. doi: 10.1186/s13007-021-00738-1 (PMC8017846; doi:10.1186/s13007-021-00738-1)
Supplement: Supplementary file 5 — Additional file 5. Non-normalized recovery, matrix effect and process efficiency. [file 13007_2021_738_MOESM5_ESM.pdf]

Additional file 5. Non-normalized recovery (RE), matrix effect (ME), and process efficiency (PE). 5-20 mg fresh weight of tissue were extracted in ice cold 10% methanol acidified with 0.1% formic acid, spiked with 10 pmol of KAR_1 and KAR_2, and analysed by UHPLC-MS/MS after purification by one-step SPE purification. The absolute peak areas obtained for neat solution standards, the corresponding peak areas for standards spiked into plant extracts after purification into plant extracts and peak areas for standards spiked before the SPE step were used to calculate the non-normalized parameters. Values are means ± SD (n = 3).

**Compounds**	**Sample weight**	**RE [%]**	**ME [%]**	**PE [%]**
KAR_1	5 mg	86 ± 30	32 ± 5	27 ± 5
	10 mg	102 ± 23	28 ± 4	27 ± 2
	20 mg	105 ± 16	25 ± 3	26 ± 1
KAR_2	5 mg	90 ± 31	57 ± 9	49 ± 8
	10 mg	103 ± 28	50 ± 9	49 ± 4
	20 mg	100 ± 23	50 ± 6	48 ± 6
