# Supplementary material for: A stable isotope dilution method for a highly accurate analysis of karrikins
Source: Plant Methods. 2021 Apr 1;17:37. doi: 10.1186/s13007-021-00738-1 (PMC8017846; doi:10.1186/s13007-021-00738-1)
Supplement: Supplementary file 6 — Additional file 6. Internal standard-normalized recovery, matrix effect and process efficiency. [file 13007_2021_738_MOESM6_ESM.pdf]

Additional file 6. Internal standard-normalized recovery (RE), matrix effect (ME) and process efficiency (PE). Values are means ± SD (n = 3). 0-20 mg fresh weight of tissue were extracted in ice cold 10% methanol acidified with 0.1% formic acid, spiked with 10 pmol of KAR_1, KAR_2 and $[^2H_3]KAR_1$, and analysed by UHPLC-MS/MS after purification by one-step SPE purification. The RA, ME and PE values were calculated based on concentrations of KAR compounds quantified by the standard isotope dilution method combined with calibration curves without (Cal 1) and with (Cal 2 and 3) plant matrix. Cal 1 – solvent-only calibration in methanol, Cal 2 – calibration dissolved in the plant matrix blanks obtained after the SPE step, and Cal 3 – matrix-matched calibration prepared similarly to the sample according to developed purification protocol. Values are means ± SD (n = 3).

**Compound**	**Sample weight**	**Non-matrix Calibration**		**Matrix Calibration**			
		**Cal 1**		**Cal 2**		**Cal 3**	
		**ME [%]**	**PE [%]**	**ME [%]**	**PE [%]**	**ME [%]**	**PE [%]**
KAR_1	5 mg	98 ± 2	102 ± 8	98 ± 2	102 ± 8	88 ± 2	97± 8
	10 mg	96 ± 3	101 ± 7	96 ± 3	101 ± 7	87 ± 3	90 ± 6
	20 mg	90 ± 2	98 ± 4	90 ± 2	98 ± 4	89 ± 2	92 ± 4
KAR_2	5 mg	145 ± 5	144 ± 20	147 ± 5	146 ± 20	120 ± 4	122 ± 17
	10 mg	150 ± 12	161 ± 10	153 ± 12	164 ± 11	110 ± 9	113 ± 7
	20 mg	139 ± 16	141 ± 19	141 ± 16	143 ± 20	108 ± 12	107 ± 15
